# Optimal Input Representation in Neural Systems at the Edge of Chaos

**DOI:** 10.3390/biology10080702

**Published:** 2021-07-23

**Authors:** Guillermo B. Morales, Miguel A. Muñoz

**Affiliations:** Departamento de Electromagnetismo y Física de la Materia, Instituto Carlos I de Física Teórica y Computacional, Universidad de Granada, E-18071 Granada, Spain; guillermobm@onsager.ugr.es

**Keywords:** information processing, input representation, neural networks, criticality hypothesis, edge of chaos, reservoir computing

## Abstract

**Simple Summary:**

Here we show that a simple neural network within the paradigm of reservoir computing is able to reproduce an important feature of internal representations of neural inputs, in agreement with what theoretically predicted and empirically measured in the mouse visual cortex, only when it is set to operate at the edge of chaos.

**Abstract:**

Shedding light on how biological systems represent, process and store information in noisy environments is a key and challenging goal. A stimulating, though controversial, hypothesis poses that operating in dynamical regimes near the edge of a phase transition, i.e., at criticality or the “edge of chaos”, can provide information-processing living systems with important operational advantages, creating, e.g., an optimal trade-off between robustness and flexibility. Here, we elaborate on a recent theoretical result, which establishes that the spectrum of covariance matrices of neural networks representing complex inputs in a robust way needs to decay as a power-law of the rank, with an exponent close to unity, a result that has been indeed experimentally verified in neurons of the mouse visual cortex. Aimed at understanding and mimicking these results, we construct an artificial neural network and train it to classify images. We find that the best performance in such a task is obtained when the network operates near the critical point, at which the eigenspectrum of the covariance matrix follows the very same statistics as actual neurons do. Thus, we conclude that operating near criticality can also have—besides the usually alleged virtues—the advantage of allowing for flexible, robust and efficient input representations.

## 1. Introduction

Understanding how the brain of mammals, including humans, represents, processes and stores information is one of the main challenges of contemporary science. In addition to the obvious direct interest in such an ambitious goal, any progress made towards elucidating the brain working principles would also help to develop a new generation of artificial intelligence devices. Reversely, advances in computer science help shedding light on the analogies and differences between our present operational knowledge on “artificial intelligence” and “natural intelligence”. This two-sided dialogue is hoped to guide exciting breakthroughs in the next coming years in both fields.

A popular idea, coming from the world of artificial neural networks [1,2] and then exported to the realm of biological systems (see [3,4] and refs. therein), is that information-processing complex systems, composed of many individual interacting units, are best suited to encode, respond, process, and store information if they operate in a dynamical regime nearby the critical point of a phase transition, i.e., at the edge between “order” and “disorder” [3,4,5,6,7,8,9,10,11,12]. In a nutshell, one can say that “ordered phases” encode information in a robust or stable way, but they are not flexible enough as to accommodate for or respond to input changes; on the other hand, “disordered phases” are dominated by noise, thus hindering information storage and retrieval. Therefore, there needs to be some kind of trade-off between order and disorder; this can be formulated in a number of different ways, e.g., between “stability and responsiveness” or between “robustness and flexibility”. The criticality hypothesis poses that such a trade-off is best resolved near criticality or “at the edge of chaos”, where combined advantages from the two alternative phases can be obtained [3]. Furthermore, at critical points there is a concomitant scale invariance—with its characteristic power-law distributions and scaling—entailing the existence of broadly different time and length scales, which seem much convenient for the representation of multiscale complex inputs. Let us remark, that the terms “criticality” and “edge of chaos” are sometimes used indistinctly, though the last one applies to deterministic systems, in which a transition occurs between ordered and chaotic states, but as recently emphasized, they can describe two sides of the same coin (we refer to [13] for an illuminating recent discussion).

Empirical evidence that actual neural networks might operate close to criticality has kept accumulating in recent years [10,14,15,16,17]. Most of this evidence (though not all) relies on the concept of neuronal avalanches [14] which are empirically observed to be scale-invariant across species, brain regions, resolution levels, and experimental techniques [3,9,18]. However, as of today, smoking-gun evidence is still needed to validate or dismiss this fascinating conjecture, and it remains controversial [19]; thus, novel theoretical and data-oriented analyses are much needed [3].

In a seemingly unrelated work, Stringer et al. have recently made a step forward in understanding how neuronal networks actually represent complex inputs [20]. In particular, these authors proved mathematically that the statistics of spiking neurons representing external sensory inputs (such as natural images represented in the mouse visual cortex) need to obey certain constraints for the input representation (or “neural code”) to be “continuous and differentiable” (henceforth *C+D*) [20]. These abstract mathematical properties are the formal counterpart of a much-desired property of neural networks: i.e., the robustness of the representation against small perturbations of the inputs. Such robustness is well-known to be sometimes violated in artificial neural networks (ANNs); in particular, so-called *adversarial attacks*, consisting in tiny variations in the input or their statistics, can fool the network, leading to wrong predictions and missclassifications [21]. We refer to Stringer et al. [20] for an in-depth explanation and justification of these important ideas, as well as to [22] for a recent application onto multi-layer ANNs. In any case, the conclusion of Stringer et al. is that, in order to achieve robust input representations, the covariance matrix of neuronal activities measured across time when the network is exposed to a sequential series of inputs must obey the following spectral property: its rank-ordered eigenvalues should decay as a power law of their rank, with an exponent α strictly larger than 1+2/d, where *d* is the embedding dimension of the input. Thus, α=1 sets a lower bound for the possible values of the eigenspectrum decay-exponent for complex, high-dimensional inputs.

Rather remarkably, these theoretical predictions have been verified to be fulfilled in experimental recordings of more than 10,000 individual neurons in the mouse visual cortex exposed to a very large sequence of natural images [20]. This confirms that information encoding occurs as mathematically predicted, i.e., in a continuous and differentiable (*C*+*D*) manifold.

The main question we pose here is: do ANNs trained to classify images encode external inputs following the same continuity and differentiability constraints found in the mouse visual cortex? More specifically, is the spectrum of eigenvalues of the associated covariance matrix a power law of the rank? Is the exponent in all cases larger than (and close to) 1? If so, do the exponent values change with the dimensionality of the images in the way predicted by Stringer et al.?

Here, as a proof of concept, we analyze the neural encoding of inputs with different dimensions in a paradigmatic example of ANN: the *echo state network* [23]. This type of ANN, together with *liquid state machines* [24,25], constitute the prototype of *reservoir computing* (RC) approaches [26], a paradigm of computation that seems particularly well suited for exploiting the putative advantages of operating at the “edge of chaos” [3].

## 2. Materials and Methods

### 2.1. Model Formulation

The Echo State Network (ESN), in its original formulation, was devised by Jaeger as a flexible and easy-trainable recurrent neural network for time-series prediction tasks [23,27]. More specifically, the architecture of ESNs consists of:

- An input layer, which scales a number $L_{1}$ of inputs at each time step before they arrive in the reservoir, according to some random weights $W^{in}\in R^{N\times L_{1}}$.
- A reservoir consisting of *N* internal units connected with random weights $W^{res}\in R^{N\times N}$, whose corresponding states evolve according to a non-linear, time-discrete dynamical equation under the influence of a time-dependent input. In this way, the reservoir maps the external input into a high-dimensional space.
- An output layer, with trainable weights $W^{out}$ that converts the information contained in the high-dimensional states of the neurons (the internal representation of the inputs) to generate the final output.

Thus, unlike in other ANNs, the internal weights or “synaptic connections” in ESNs do not need to be updated during the learning process, and training is achieved by just modifying the layer of output weights that read out the network internal states.

**Figure 1:**
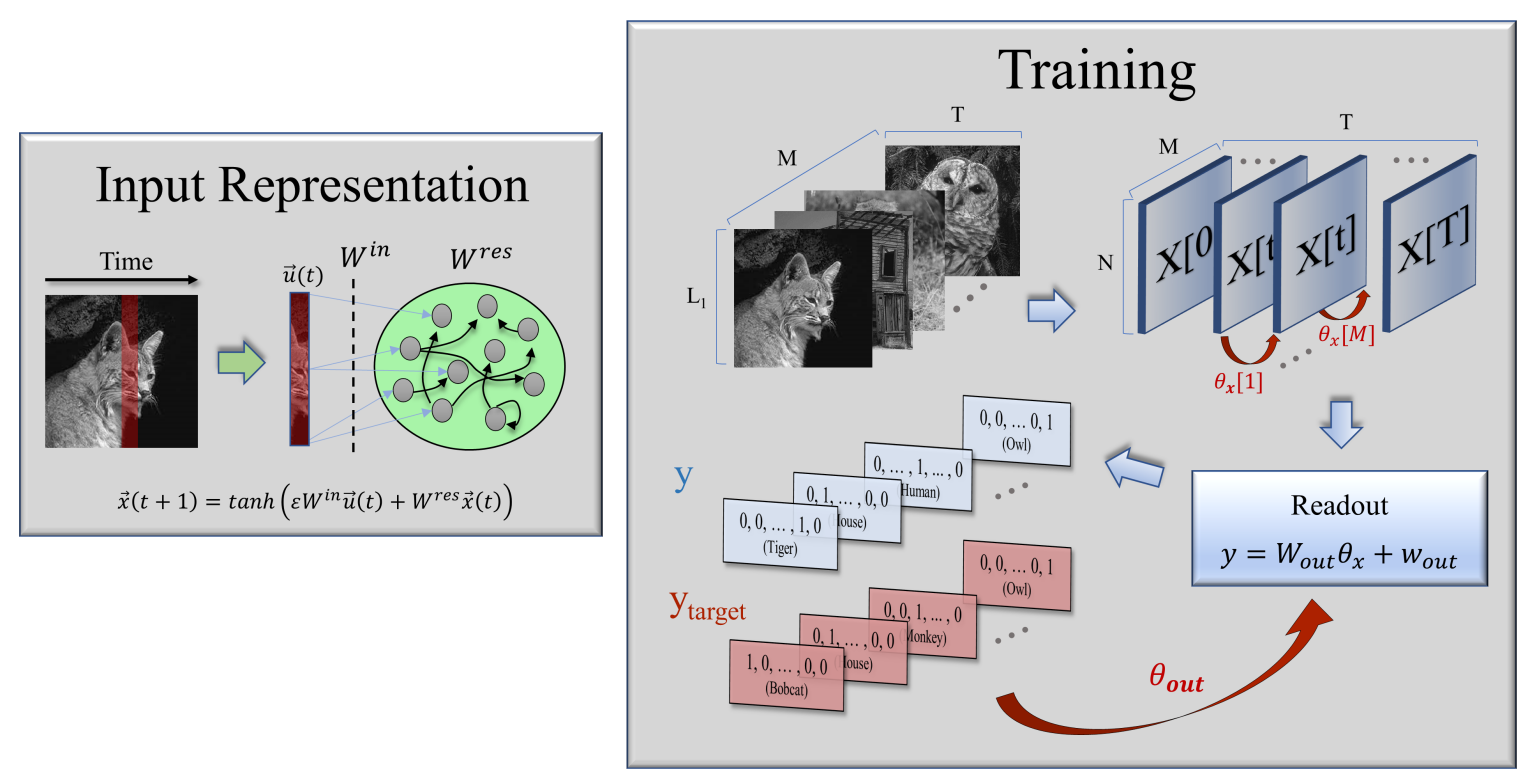
Sketch of the echo state network and the image classification task. **Left**: Images are converted to multivariate time series and then fed into the reservoir. **Right**: for each processed image a set of parameters $\theta_{x}$ is generated, which characterizes the high-dimensional state of the reservoir, i.e., the “*reservoir model space*”. These are then fed into the readout module, which linearly transforms the information in the reservoir model space into an output label. Finally, output weights ${\tilde{W}}_{out}$ are generated by minimizing the error between the predicted and target labels. Red arrows indicate steps in which a Ridge regression is performed.

To adapt this architecture—usually employed in time-series analyses—for image classification tasks, we used black and white images with $L_{1}\times L_{2}$ pixels (each of them characterized by a value in the [0,1] interval, representing a normalized grey-scale) and converted them into multivariate time series by considering their vertical dimension as a vector of $L_{1}$ elements or features, that “evolve” along $T=L_{2}$ discrete “time” steps. One can then define a standard training protocol in which, as illustrated in Figure 1, at each time t∈[0,T], vectors $u\left(t\right)\in {\left[0,1\right]}^{L_{1}}$ corresponding to columns of a given image are fed as inputs to the ESN. In this way, the network dynamics for the reservoir states is given by the following non-linear activation function:

(1) $$x\left(t\right)=\tanh(\varepsilon W^{in}u\left(t\right)+W^{res}x\left(t-1\right))$$

where ε is an overall input scaling factor.

Using a supervised learning scheme, the goal of the ESN is to generate an output label $y\in N^{F}$ that correctly classifies each image in the test set as belonging to one of the *F* existing categories or classes (e.g., “bobcat”, “owl”, etc. for the illustration in Figure 1). This label consists of a vector in which every element is zero, except for a value of one at the position corresponding to the assigned class (i.e., “one-hot-encoded” in the machine learning jargon). Several readout methods have been proposed in the literature to transform the information contained in the reservoir dynamics into the expected target output $y^{target}\in N^{F}$, ranging from linear regressions methods over the reservoir states [28,29], to the use of “support vector machines” or “multilayer perceptrons as decoders [30]”. Here, we use a simple Ridge regression (see Appendix A for a detailed explanation of the algorithm) over the *“reservoir model space”*, a method that has been recently proposed for the classification of multivariate time series [31].

The reservoir model space is a set of parameters $\theta_{x}$ that encodes the network *dynamical* state for a given input (image). Such parameters are obtained from a Ridge regression to predict the next reservoir state from the past one at discrete time steps,

(2) $$x\left(t+1\right)=W_{x}x\left(t\right)+w_{x},$$

in such a way that $\theta_{x}=\left[\mathrm{vec}\left(W_{x}\right);w_{x}\right]\in R^{N\left(N+1\right)}$ provides a characterization of the internal reservoir dynamical state during the presentation of a given input, where vec(·) denotes reshaping to a one-column vector and “;” vertical concatenation. Then, for each image, a readout module or decoder can transform this internal representation into an output label:

(3) $$y=W^{out}\theta_{x}+w_{out}.$$

The parameters $\theta_{out}=\left[\mathrm{vec}\left(W_{out}\right);w_{out}\right]$ —where $W^{out}\in R^{F\times N(N+1)}$ and $w_{out}\in R^{F}$ are defined as output weights and biases, respectively— are determined again through Ridge regression, minimizing the error between the produced and target label for all the presented images in the training set.

Let us remark that the presented framework can be naturally extended to include, for instance, leakage and noise terms in Equation (1), feedback connections from the output to the reservoir, or plastic rules that modify the reservoir weights according to the inputs [32,33], among other possible extensions. However and since our aim here is not to reach state-of-the-art classification accuracy—but rather highlight the link between optimal input representation and the internal dynamical state—we refrain for the sake of parsimony from adding further features to our model, leaving these potential extensions for future work.

### 2.2. Image Datasets and Parameter Selection

**Figure 2:**
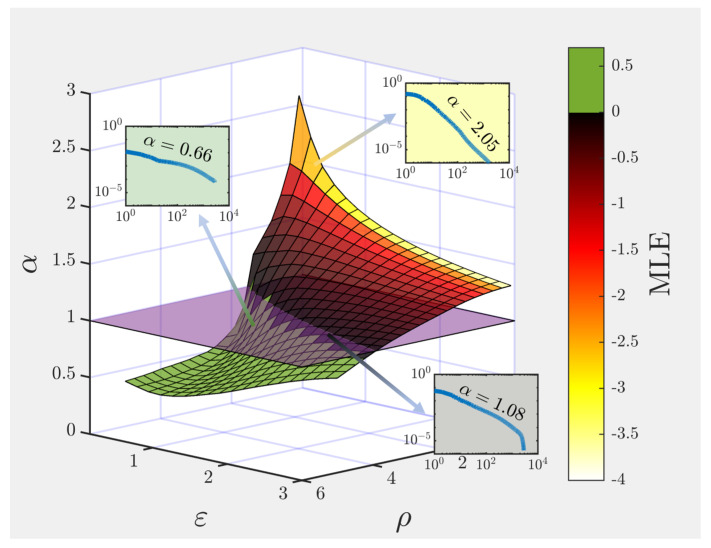
Exponent for the power-law decay of the spectrum of the activity covariance matrix as a function of the spectral radius (ρ) and input scaling factor (ε) of the reservoir, plotted together with the maximum Lyapunov exponent (MLE) color-coded within the surface. The insets correspond to the activity covariance matrix eigenspectrum measured in three different points of the parameter space, where the variance in the *n*-th dimension (*n*-th eigenvalue) scales as a power-law $n^{-\alpha }$ of the rank. For ease of visualization, the plane separating the region α<1 in which the representation is no longer C+D was plotted in purple.

**Figure 3:**
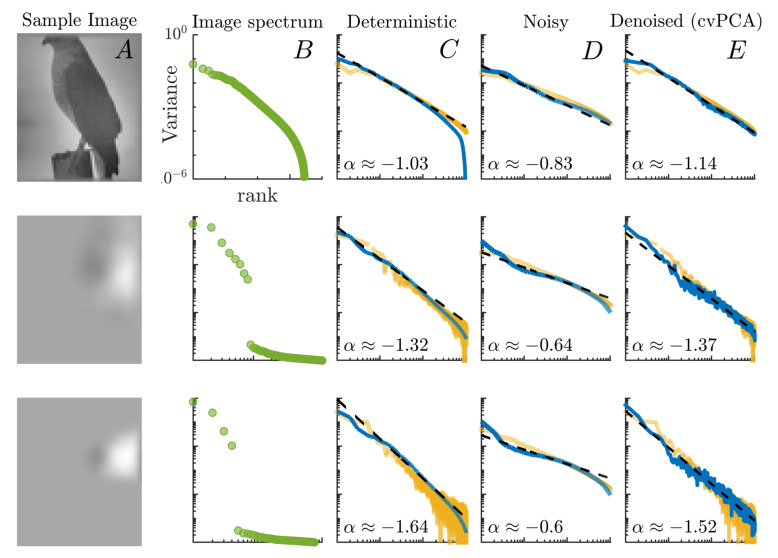
From left to right: (**A**) sample from the M=2800 images in the training set; (**B**) eigenspectrum of the images pixel intensities; (**C**) eigenspectrum for the activities of an echo state network (blue line) and actual, V1 mouse neurons (yellow line, plotted after [20] applying cross-validated PCA; see Materials and Methods) when subject to images of dimensionality *d*; (**D**) same analysis, but now zero-centered white noise of amplitude ξ=0.4 is added to the neuron dynamics (blue line), and no cvPCA is performed over the experimental values (yellow line); (**E**) same analysis as in (**D**), but now noise has been subtracted using cvPCA. From top to bottom: results for natural, high-dimensional images; the same images projected onto 8 dimensions; the same images projected onto 4 dimensions. To obtain the ESNs eigenspectra, parameters were chosen so that the networks operated near the edge of chaos, with $\lambda ∼-5\times 10^{-3}$.

For the first part of the results (Figure 2 and Figure 3), we used the same set of M=2800 natural images from the ImageNet database [34] that were employed in the experiments of Stringer et al. [20]. Four and eight-dimensional stimuli constructed from a reduced-rank regression model over the original images (see Figure 3) were also extracted from the data used [20], publicly available at [35]. ESNs were constructed with N=2000 units, while exploring different values for the largest eigenvalue (spectral radius) of the reservoir weight matrix $W^{res}$ and input scaling factor ε.

For the image classification task in the second part of the results, we resort to the canonical MNIST dataset which includes 60,000 handwritten instances of the first 10 digits as training and 10,000 for testing. Due to the large computational cost of storing the reservoir model space for each image, the ESNs were trained in only one-third of the training set but validated in the full testing set.

Throughout all the results, the density of the reservoir-weight-matrix elements (i.e., the percentage of non-zero connections) is kept fixed to 10%, while both reservoir and input weights are extracted at random from a uniform distribution in the interval [−1,1].

### 2.3. PCA and cvPCA

**Figure 4:**
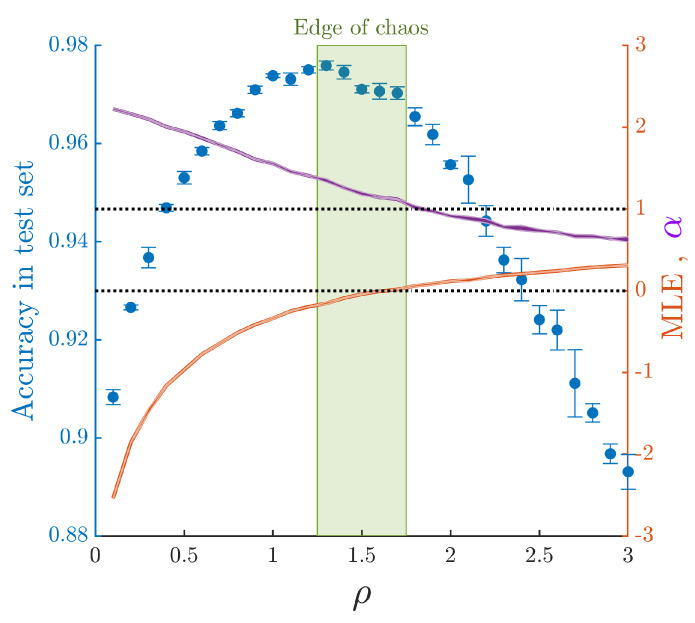
Curves for the accuracy in MNIST testset (blue dots), maximum Lyapunov exponent (orange line) and best-fit exponent for a power-law spectrum of the activity covariance matrix (purple line). Training was performed over 20,000 randomly chosen images of the MNIST training dataset, while classification error was assessed over the full test set (10,000 images). Errors in each case were estimated as the standard deviation from the mean over ten different initializations of the ESN.

Following the same methodology as Stringer et al. [20], to obtain the results in Figure 2, Figure 3B–D and Figure 4, the ESNs were first presented with the corresponding set of input images, and the activity of the internal units in the reservoir was stored for each step of the training. Then, principal component analysis (PCA) was performed directly over the full set of neuronal activities $X\in R^{N\times (T\times M)}$, where *T* is the number of pixels in the horizontal dimension of the images (T=90 for natural, four and eight-dimensional images; T=28 in the MNIST dataset). In this way, we obtained the variance (i.e., the associated eigenvalue) along each principal component or eigenvector of the covariance matrix, which serves in turn as a basis for the activity inside the reservoir (see [36] for a very gentle but rigorous introduction to PCA). Eigenvalues were then rank-ordered and fitted to a power-law using the approach developed by Clauset et al., which combines fitting methods based on maximum likelihood with tests for the goodness of the fit based on the Kolmogorov–Smirnov statistic and likelihood ratios. For a careful explanation of the method and implementation details we refer to [37].

To analyze the effects of noise and trial-to-trial variability in real experiments, we also studied a model of ESN including a white-noise term of amplitude ξ = 0.4 inside the activation function (Figure 3C). Then, we used the same cross-validated PCA method proposed teStringer to generate an unbiased estimate of the signal (or input-related) PC variances. In short, the stimulus-related variance confined in an n-dimensional manifold can be extracted by first computing the eigenvectors spanning this manifold from a first repeat of the full training set and then measuring the amount of a second repeat’s variance that is confined to this plane (we refer to [20] for a detailed explanation and derivation of the cvPCA method).

## 3. Results

### 3.1. Non-Trivial Scaling and Robust Input Representation at the Edge of Chaos

Although relatively simple, our proposed ESN model has several hyperparameters that can be tuned, affecting its performance. More specifically, the *spectral radius* ρ of the reservoir internal weight matrix and the *scaling factor*ε of the input weights are two variables that usually determine the dynamical regime within the reservoir [26]. The spectral radius —or largest eigenvalue of the reservoir weight matrix $W^{res}$—controls the dynamical stability inside the reservoir when no input is fed into the network. Thus, a spectral radius exceeding unity has been often regarded as a source of instability in ESNs due to the loss of the so-called “*echo state property*”, a mathematical condition ensuring that the effect of initial conditions on the reservoir states fades away asymptotically in time [23,38,39]. Nevertheless, later studies have shown that the echo state property can be actually maintained over a unitary spectral radius, and different sufficient conditions have been proposed [39,40,41] (see in particular [42], where the authors analyze the problem from the lens of non-autonomous dynamical systems, deriving a sufficient condition for the echo state property with regard to a given input). On the other hand, increasing the value of ε can convert an initially expanding mapping into a contracting dynamics, as stronger inputs tend to push the activities of the reservoir units towards the tails of the non-linearity.

In what follows, we analyze the input representation that the reservoir codifies in terms of the trade-off between ρ and ε, which *together* determine the dynamical operating regime of the ESN and the presence or absence of the echo state property. Notably, we find that the spectrum of covariance matrix eigenvalues as a function of their rank (i.e., the variance associated to the *n*-th principal component, when ordered from the largest to the smallest) can be well fitted to a power-law $n^{-\alpha }$ (see insets in Figure 2), whose associated exponent α decays (flatter spectrum) with the spectral radius ρ; while increases (faster decay) with the input scaling factor ε for most of the parameter space (see Figure 2). In [20] it was found that the exponent of this power-law relation is close to 1 when natural, high-dimensional images were shown to the mouse as an input. As discussed above, the authors proved mathematically that α>1+2/d is a necessary condition for the neural manifold that emerges from the representation of a *d*-dimensional input to be C+D. Additionally, if α<1, the representation is not continuous nor differentiable, and if 1<α<1+2/d the representation can be continuous but not differentiable. For natural images, *d* is very large and one can approximate the critical exponent by $\alpha_{c}\approx 1$ (a condition that is marked by the purple plane in Figure 2).

As the following step, one might naturally wonder if there is some aspect of our model that characterizes such a regime of robust representations in the parameter space (ρ,ε) that controls the dynamical state, for which an exponent α close to unity is found. In other words: is the dynamics of the system inherently different in the regions for which the input representation manifold is found to be C+D (i.e., above the purple plane in Figure 2)?

As it turns out, the exponent α characterizing the decay of the eigenspectrum approaches unity for choices of ρ and ε that drive the network dynamics towards the so-called “edge of instability” or “edge of chaos”, this is, near a transition point between an ordered and a chaotic regime (see Figure 2). Traditionally, chaotic regimes are characterized by their average sensitivity to perturbations in the initial conditions; to quantify this effect, one usually measures the rate of divergence of two trajectories with a very small difference in their initial conditions:

(4) $$\lambda =\underset{k\to \infty }{lim}\frac{1}{k}log\left(\frac{\gamma_{k}}{\gamma_{0}}\right)$$

where λ is termed the maximum Lyapunov exponent (MLE), $\gamma_{0}$ is the initial distance between the perturbed and unperturbed trajectories, and $\gamma_{k}$ is the distance between the trajectories at step *k* (we refer the reader to [43,44] for a detailed explanation of the algorithm used to compute the MLE). Thus, chaotic dynamics are typically associated with a positive MLE, while the system is said to be stable to local perturbations provided λ<0. It can be clearly seen from Figure 2 that the region in which one finds non-C+D representations of the input (below the purple plane) matches almost perfectly with the region (colored in green) in which a positive MLE is found.

**Figure A1:**
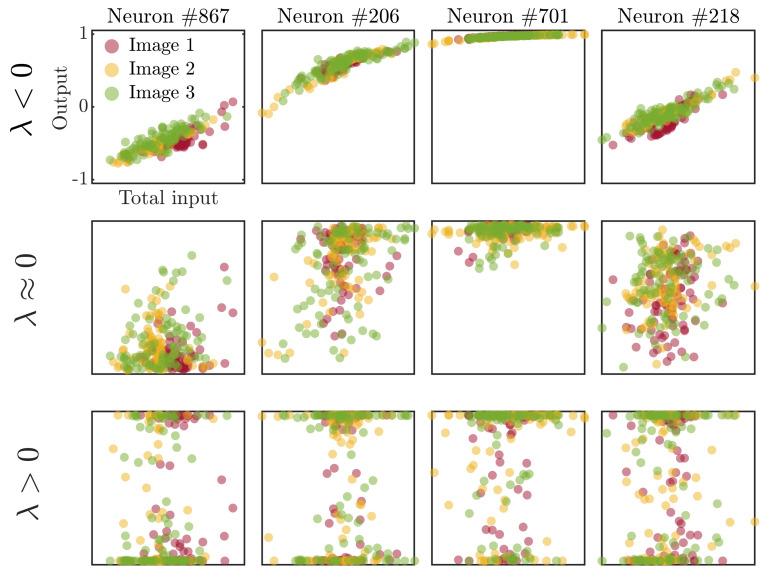
Activity of four different neurons operating in the sub-critical, critical and super-critical regimes (from top to bottom) when presented with three different high-dimensional, natural images. Each point in the panels represents the activity $x_{i}\left(t\right)$ of neuron *i* at a different time step *t* as a function of the total input $f_{i}\left(t\right)=\varepsilon \sum_{l=1}^{L_{1}}w_{il}^{in}u_{l}\left(t\right)+\sum_{j=1}^{N}w_{ij}^{res}x_{j}\left(t-1\right)$ arriving to it.

The transition order-to-chaos can be also visualized by looking directly at the activities inside the reservoir (see Figure A1). Observe that, when the network is in an “ordered” state, with λ<0, the responses of the neurons are quite heterogeneous when compared among them, but they are highly localized within each neuron, i.e., individual neurons experience a limited response to stimuli. On the other hand, dynamical states characterized by λ>0 have neurons whose response extends across the full range of the non-linearity (with higher probability along with the tails, reflecting a saturated behavior), but it is this same “phase space expansion” that makes units almost indistinguishable from each other. It is only around the critical point or edge of chaos, that we find a compromise between dynamical richness in individual units and variability across them.

Coming back to the results in Stringer et al., one may also wonder whether the continuity and differentiability condition α>1+2/d holds also for low-dimensional inputs, for which the expected bound $\alpha_{c}=1+2/d$ deviates considerably from unity. To this purpose, Figure 3 shows the measured eigenspectrum of the reservoir activity covariance matrix (i.e., eigenvalues as a function of their rank) when images of different dimensionality (the same ones used by Stringer *et al.* in their experiments) are presented as inputs, and the reservoir is tuned to operate at the onset of a chaotic regime, i.e., for values in the parameter space $\left(\rho ,\varepsilon \right)$ for which λ was near zero but still negative. Remarkably, we find in all cases that *the exponents observed in the mouse visual-cortex activity are best reproduced when the reservoir dynamics is tuned close to the “edge of chaos”*.

This finding suggests that one can set the network parameters in such a way that the neural activity manifold in which the input is represented is almost as high-dimensional as possible without losing its “smoothness”, and that such optimal solution is found at the edge of chaos.

At this point, it is pertinent and timely to dig a bit deeper into the similarities and differences between the results presented [20] for real, V1-cortex neurons in the mouse, and the power-law exponents obtained through our reservoir computing model.

(i) First of all, as in the case of real neurons, the observed correlations between the internal units are not just a byproduct emerging from scale-free features of natural images (see the second column in Figure 3). In particular, one can see that the power-law decay of the covariance matrix eigenspectrum persists even in response to low-dimensional inputs whose embedding vector space can be spanned with just a few principal components (i.e., lacking a power-law decaying intrinsic spectrum).
(ii) In our model, images are processed sequentially in time along their horizontal dimension so that for each image one can measure the activity of the *N* internal units over $T=L_{2}$ time steps. In contrast, the activity of V1 neurons [20] is scanned at a relatively low rate, so that for each image the neural representation is characterized by just one amplitude value in each neuron.
(iii) To avoid confusion, let us remark that the covariance matrix observed by Stringer et al. is not directly measured over the raw activity of the neurons. Instead, the author’s first project out the network spontaneous activity from the data, and then perform a cross-validated PCA (see Materials and Methods) that allows them to filter out the trial-to-trial variability or “noise”. However, as our model is completely deterministic for a given initialization of an ESN, the stimulus-related variance computed through cvPCA trivially matches that of a standard PCA.

A natural question then arises from this last point: what happens when a noise term is included inside the non-linear function in Equation (1), so that the dynamics becomes stochastic? Are the power-law exponents robust to the introduction of noise? To answer these questions, we considered stochastic versions of the ESNs—including an independent small additive noise term in their inputs—and presented them with two repeats of the same input training set. The internal states of the noisy reservoirs were collected at each time step (Figure 3D). We then performed the same type of cvPCA analyses proposed in [20] (see Materials and Methods) to estimate the signal variance in our reservoirs (Figure 3E). Just as in the case of real V1 neurons, the exponents measured over the raw, noisy activity are lower and below the critical threshold for C+D of the neural manifold. Nevertheless, a cvPCA over the internal states retrieves the expected exponents after noise has been filtered out.

We will further comment on the possible implications of this finding in the Discussion section, but for now, let us wrap up our findings tackling what we believe is a fundamental question from the perspective of machine learning: does working at the edge of chaos (or, equivalently, having optimal, C+D neural manifolds) provide any functional advantage?

### 3.2. Solving a Benchmark Classification Task

The advantages of working at the so-called edge of chaos were first pointed in general dynamical systems and cellular automata [1,45], and only later analyzed in reservoir computer models with binary [46,47,48] and analog [43,48] internal units. In particular, in [43] the authors showed that ESNs presented maximal information storage and transfer, as well as enhanced memory capacity right at the edge of chaos. However, while ESNs and other RC approaches have been previously applied to classification tasks with very good results [31,49,50,51,52,53], to the best of our knowledge, an analysis of the influence of the dynamical regime on the performance of RC architectures for classification tasks is still missing.

As a proof of concept, here we measure the performance of ESNs in a simple classification task over a subset of the canonical MNIST dataset. The results, shown in Figure 4, highlight the fact that optimal performance (∼2.2% error rate) is found just below the onset of chaos when λ≲0. Most notably, the plot also evinces that the decay in performance is not only preceded by a positive MLE but coincides too with exponents α for the fit of the covariance-matrix eigenspectrum that are below the limiting value $\alpha_{c}\approx 1$, indicating the loss of C+D property of the neural representation manifold for high-dimensional images. While the results presented here are for a fixed value of the input scaling ε=0.6, exploratory simulations seem to confirm that the main results remain unchanged for other values of ε. A more systematic analysis exploring in detail all the phase space as well as other datasets containing natural images such as CIFAR-10 or ImageNet will be published elsewhere.

We finally remark that these results were obtained with a reservoir consisting only of 500 internal units and using only one-third of the training set, with no pre-processing of the images. In contrast, the current best performance in MNIST digit recognition (0.81% error rate) using reservoir computing networks has been achieved with a two-layer architecture, each consisting of 16,000 units, which amounts to a total of 880,000 trainable parameters [54]. In this sense, when compared to ESNs with a greater number of units and much more complicated dynamics trained over the full MNIST dataset [49], our simple ESN with readouts over the reservoir model space shows comparable performance when tuned near the edge of chaos.

## 4. Conclusions and Discussions

The main contribution of this work is to show that a simple model of ANN does generate optimal and robust input representations (i.e., obeying the conditions for continuity and differentiability derived by Stringer et al. [20] when it operates close to an edge-of-chaos type of critical point. To this aim, we have shown that echo state networks composed of randomly-connected units, when subject to an external input, are able to reproduce power-law exponents similar to those found in mouse V1-cortex for the decay of the covariance matrix eigenspectrum. Most notably, adding stochasticity in the form of small-amplitude white noise inside the reservoir dynamics leads to flatter eigenspectra, much like those found in raw experimental data. This result therefore suggests that the role of spontaneous intrinsic activity and trial-to-trial variability on the representation of external inputs can be easily accounted for in our simple ESN model.

Let us remark that, the slower the decay (i.e., the larger the exponent) the more weight is given to fine details of the input, but if the decay is too slow (smaller than the lower bound given by α=1+2/d), an excessive importance is given to such fine details at the cost of hampering the existence of a “smooth manifold” representation. Thus, operating near the edge of chaos seems to provide the network with an optimal trade-off between representing as much details as possible and constructing operative, smooth representations, which we have shown translates into an improved performance in image classification tasks.

We find nevertheless important to clarify that the term edge of chaos—and the concept of chaos itself—should be taken with caution as it is not devoid of criticism in this context. As pointed out in [42], ESNs are an example of non-autonomous dynamical systems, for which typical concepts based in the theory of autonomous systems (e.g., “sensitivity to initial conditions”, “attractor” and “deterministic chaos”) do not directly apply [55,56]. In fact, the authors of [42] claim that local perturbation experiments cannot represent an ultimate evidence of chaotic dynamics in non-autonomous systems, since it might well be the case that the input drives the system towards and expanding dynamics for a certain time span, while the system shows on average a contracting, non-chaotic dynamics. Despite these caveats, at the light of the presented results it appears like there is indeed an actual dynamical phase transition occurring as the maximum Lyapunov exponent crosses zero. Thus, in any case, it seems a sensible choice to use such a quantity as a control parameter when analyzing the underlying neural representation of external inputs.

Therefore, the presented results open the path for very exciting research avenues at the boundary of biology and machine learning, calling for theoretical formulations that can shed light into the fascinating properties of these input-representation neural manifolds and their relation with the criticality hypothesis.

## Data Availability

No new data have been used.

## Appendix A. Ridge Regression

Readouts in ESNs are typically linear, as it can be seen from Equation (3). To simplify the forthcoming derivations, let us rewrite Equation (3) as:

(A1) $$y={\tilde{W}}^{out}\left[\theta_{x};1\right]$$

where ${\tilde{W}}^{out}\in R^{F\times \left(N(N+1)+1\right)}$ and ";" indicates vertical vector concatenation. Thus, for a given input image a reservoir representation of the states $\theta_{x}$ is constructed, and one can use the above equation to generate the corresponding output (which in our case will be a one-hot-encoded label $y\in N^{F}$ classifying the image). We can trivially generalize the above equation to apply to the full set of *M* images in the training set $Y\in N^{F\times M}$:

(A2) $$Y={\tilde{W}}^{out}\Theta_{x}$$

where $\Theta_{x}\in R^{\left(N\left(N+1\right)+1\right)\times M}$ contains as columns the vectors $\left[\theta_{x};1\right]$ generated for each of the *M* input images. Finding the optimal weights ${\tilde{W}}^{out}$ that minimize the squared error between the produced and target labels, y and $y^{target}$, is then reduced to a standard linear regression problem, which is the greatest strength of the reservoir-computing approach:

(A3) $$Y^{target}={\tilde{W}}^{out}\Theta_{x}.$$

Owing to the fact that large output weights are commonly associated to over-fitting of the training data [32], it is a common practice to add a regularization term to the error in the target reconstruction, usually defined in terms of the root-mean-squared error (RMSE). Although several methods have been proposed to achieve this regularization [28,29,32], one of the most efficient and stable algorithms is Ridge regression, which aims to solve: -4.6cm0cm

(A4) $${\tilde{W}}^{out}=\underset{\left\{{\tilde{W}}_{out}\right\}}{\arg\min}\frac{1}{M}\sum_{n=1}^{M}\sum_{i=1}^{F}{\left(y_{i}\left[n\right]-y_{i}^{target}\left[n\right]\right)}^{2}+\beta {∥{\tilde{w}}_{i}^{out}∥}^{2}=Y^{target}\Theta_{x}^{T}{\left(\Theta_{x}\Theta_{x}^{T}+\beta I\right)}^{-1},$$

where $∥\cdot ∥$ stands for the Euclidian norm, *I* is the identity matrix and β is the regularization coefficient. Notice that choosing β=0 removes the regularization, turning the Ridge regression into a standard generalized linear regression problem (we used β=1 across all simulations in the paper).

Let us finally remark that, in order to obtain the reservoir model space parameters $\Theta_{x}$, the same Ridge regression is also implemented to solve Equation (2) for each of the input images.

## Appendix B. Phase Space of Reservoir Units

In Figure A1, we reproduce the phase space of 4 different internal units of a reservoir operating (from top to bottom lines) in a sub-critical, critical and super-critical regimes, respectively. Each plot represents the activity of the corresponding neuron at each time step against the total input (sum of external input plus reverberating activity in Equation (1)) arriving to the neuron at the previous time. Since each image is first transformed into a multivariate time series of $T=L_{2}=90$ time steps—and we plot the activity along the first three images only—each panel in Figure A1 contains 270 points.
